# Variability of transient elastography-based spleen stiffness performed at 100 Hz

**DOI:** 10.1186/s41747-023-00393-2

**Published:** 2023-12-12

**Authors:** Angelo Armandi, Talal Merizian, Merle Marie Werner, Harvey O. Coxson, Tiziana Sanavia, Giovanni Birolo, Isabella Gashaw, Judith Ertle, Maurice Michel, Peter R. Galle, Christian Labenz, Tilman Emrich, Jörn M. Schattenberg

**Affiliations:** 1 Division of Gastroenterology and Hepatology, Department of Medical Sciences, University of Turin, Corso Dogliotti 14, Turin, 10126 Italy; 2 Metabolic Liver Disease Research Program, I. Department of Medicine, University Medical Center of the Johannes Gutenberg-University, Langenbeckstrasse 1, Mainz, 55131 Germany; 3 Boehringer Ingelheim Pharma GmbH & Co. KG, Biberach & Ingelheim, Germany; 4 Computational Biomedicine Unit, Department of Medical Sciences, University of Turin, Corso Dogliotti 14, Turin, 10126 Italy; 5 Boehringer Ingelheim International GmbH, Ingelheim, Germany; 6 Department of Radiology, University Medical Center of the Johannes Gutenberg-University, Langenbeckstrasse 1, Mainz, 55131 Germany

**Keywords:** Elasticity imaging techniques, Hypertension (portal), Liver cirrhosis, Reproducibility of results, Spleen

## Abstract

**Background:**

Spleen stiffness measurement (SSM) performed by transient elastography at 100 Hz is a novel technology for the evaluation of portal hypertension in advanced chronic liver disease, but technical aspects are lacking. We aimed to evaluate the intraexamination variability of SSM and to determine the best transient elastography protocol for obtaining robust measurements to be used in clinical practice.

**Methods:**

We analyzed 253 SSM exams with up to 20 scans for each examination, performed between April 2021 and June 2022. All SSM results were evaluated according to different protocols by dividing data into groups of *n* measurements (from 2 to 19). Considering as reference the median SSM values across all the 20 measurements, we calculated the distribution of the absolute deviations of each protocol from the reference median. This analysis was repeated 1,000 times by resampling the data. Distributions were also stratified by etiology (chronic liver disease *versus* clinically significant portal hypertension) and different SSM ranges: < 25 kPa, 25–75, and > 75 kPa.

**Results:**

Overall, we observed that the spleen stiffness exam had less variability if it exceeded 12 measurements, *i.e.*, absolute deviations ≤ 5 kPa at 95% confidence. For exams with higher SSM values (> 75 kPa), as seen in clinically significant portal hypertension, at least 15 measurements are highly recommendable.

**Conclusions:**

Fifteen scans per examination should be considered for each SSM exam performed at 100 Hz to achieve a low intraexamination variability within a reasonable time in clinical practice.

**Relevance statement:**

Performing at least 15 scans per examination is recommended for 100 Hz SSM in order to achieve a low intraexamination variability, in particular for values > 75 kPa compatible with clinically significant portal hypertension.

**Key points:**

• Spleen stiffness measurement by transient elastography is used for stratification in patients with portal hypertension.

• At 100 Hz, this method may have intraexamination variability.

• A minimum of 15 scans per examination achieves a low intraexamination variability.

**Graphical Abstract:**

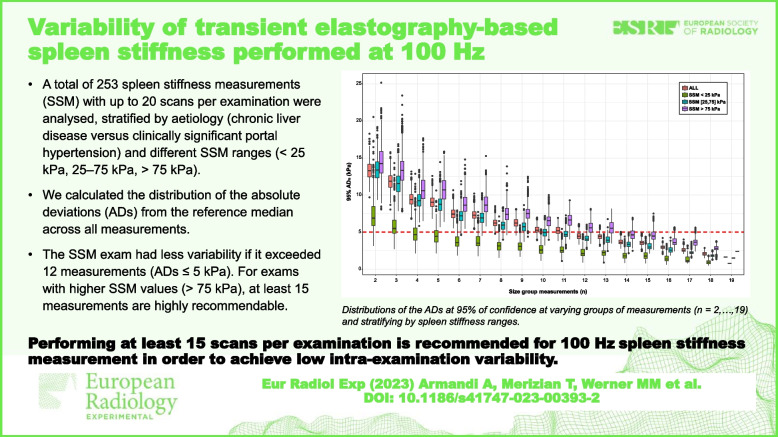

**Supplementary Information:**

The online version contains supplementary material available at 10.1186/s41747-023-00393-2.

## Background

The development of portal hypertension is a critical step in the pathogenesis of liver disease. It is responsible for clinical decompensation, including ascites, hepatic encephalopathy, and esophageal varices [1]. Importantly, the onset of portal hypertension is linked to a stepwise increase in liver-related mortality, and this clinical decompensation represents a healthcare burden that requires huge resource utilization, and it is associated with increased short-term and inpatient mortality [2–4]. The reference standard to diagnose portal hypertension is the measurement of the hepatic venous pressure gradient, an invasive procedure burdened by high costs and limited accessibility [5].

Splenomegaly is a hallmark of portal hypertension, occurring in about two-thirds of cirrhotic patients. It is often related to congestion and hypersplenism, with subsequent reduction of the circulating platelet pool. In addition, accumulation of fibrotic tissue, enhanced neoangiogenesis, and increased white pulp volume and lymphatic vessels are important structural features of this tissue hyperplasia, ultimately resulting in increased spleen stiffness [6]. These changes in size and, particularly, structure make the spleen an ideal target for quantitative non-invasive imaging procedures, similar to the approaches used to stage liver disease severity.

Over the last several years, there has been progress in the realm of non-invasive diagnostics, and transient elastography has become a part of the clinical assessment of patients with liver disease [7]. In fact, transient elastography for liver stiffness has been adopted by the latest guidelines for the stratification of asymptomatic patients with advanced chronic liver disease at risk for developing portal hypertension (Baveno VII) [8]. Moreover, recent evidence supports the use of spleen stiffness measurement (SSM) for the evaluation of clinically significant portal hypertension (CSPH) [9, 10] or even liver disease staging and treatment response [11, 12].

The early studies of SSM by transient elastography were conducted using the standard FibroScan device and a probe that generates a shear wave with a frequency of 50 Hz. However, the spleen is stiffer and located more superficially than the liver, which results in an overestimation of spleen stiffness, with measurements that are limited by the upper limits of the device [12]. The recent introduction of a novel 100 Hz spleen-specific probe has overcome some of these technical issues, and it is thought to provide more accurate SSM measurements [13, 14]. While early data suggests that the new 100 Hz spleen-specific probe is an advanced device with respect to the previous technology for SSM, there are limited reference standards or data available on the intraexamination variability. For example, the restriction of the 100-Hz technology to the M probe and the intrinsic features of the spleen parenchyma (including size and anatomic location) may affect the robustness of the results. Additionally, the manufacturer recommends performing 10 scans to obtain a reliable SSM, which is based on earlier devices and liver stiffness measurements. The operating parameters of the FibroScan device for the liver have been developed over many years, and there are numerous published studies to show the clinical utility of the measurement despite the intra- and interexamination variability in diverse populations. Nevertheless, the application of transient elastography for liver stiffness has provided evidence of clinically useful information [15–18]. Even though the 100-Hz spleen-specific probe is a novel application of this technology, there is hope that it will provide useful information on the effects of portal hypertension.

Therefore, the aims of this study were to evaluate the intraexamination variability of SSM and to determine the best transient elastography protocol to obtain reproducible measurements to use in clinical practice.

## Methods

### Study population

**Fig. 1:**
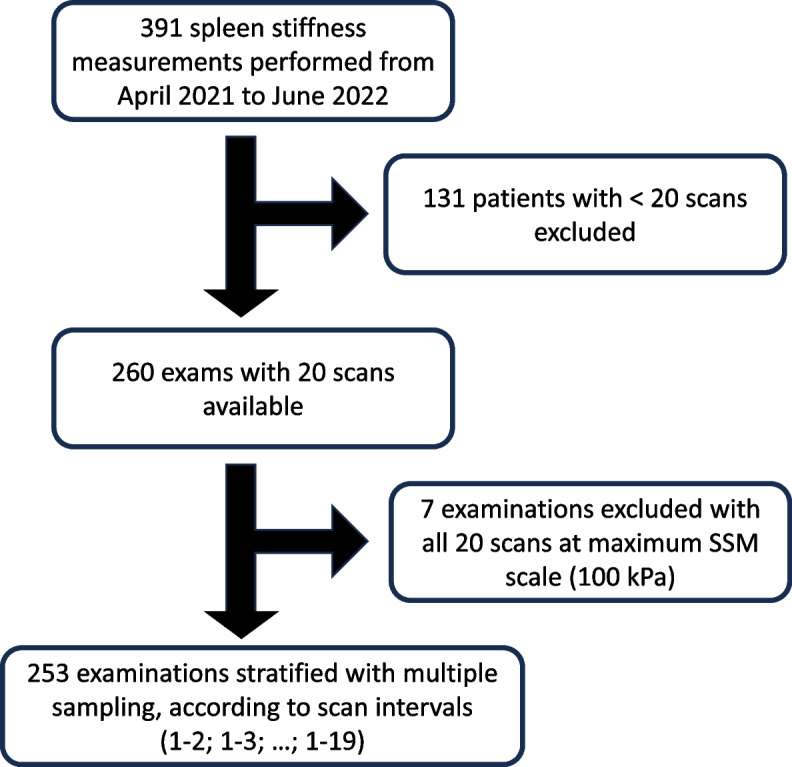
Flow chart of the spleen stiffness measurement data acquisition. SSM, Spleen stiffness measurement

A flow chart of the study is illustrated in Fig. 1. For this retrospective study, we extracted the technical data on SSM (*i.e.*, measurements in kPa, valid number of measurements, duration of the exam, calibration status, and shear wave speed in cm/s), performed at the University Medical Center of Mainz, Germany, from April 2021 to June 2022. The following anonymized clinical data were retrieved for all the examined patients: age, sex, and body mass index. All the examinations were conducted in a fasting state. In addition, indication for performing the exam was retrieved: the presence of chronic liver disease of any etiology (CLD) I presence of CSPH, which was indirectly assessed by either esophageal varices or previous liver-related event (varices bleeding, ascites, hepatic encephalopathy) or abdominal collateral circles at imaging.

SSM examinations from 391 patients were initially considered for the study; however, only examinations with 20 scans were kept for the analyses, excluding examinations from 131 patients with less than 20 scans per exam. Finally, 7 male patients, whose stiffness measurements were 100 kPa for all the 20 scans, were excluded as no meaningful analysis of variation was possible, since the exam reached a maximum scale. Written informed consent was obtained from all subjects (patients) in this study. Institutional Review Board approval was obtained, and the study protocol was approved by the ethics committee of the Landesärztekammer Rheinland-Pfalz Nr. 873.199.10 (7208). The study was conducted according to the ethical guidelines of the 1975 Declaration of Helsinki (6th revision, 2008).

### Spleen stiffness measurements

All SSM were performed using FibroScan 630 Expert device (Echosens, France, software version 4.1.2 P1 with Smart Exam) by three expert investigators (A.A., M.M.W., T.M.). The measurements were conducted using the 100 Hz shear wave frequency M-probe (3.5 MHz ultrasound center frequency; measurement depths 25–55 mm; stiffness range 6.0–100 kPa). The spleen was identified using the B-mode ultrasonography probe on the FibroScan device, and the measurements were obtained at the mean posterior axilla line between the ninth and tenth intercostal spaces with the probe positioned at the middle of the spleen.

### Statistical analysis

Clinical data of the patients’ cohort were evaluated in terms of median and interquartile range (IQR) for continuous variables and with number (%) for categorical variables. *p*-values for pairwise comparisons considering the two etiologies, *i.e.*, CSPH and CLD, correspond to the Kolmogorov–Smirnov, Wilcoxon rank-sum, and *χ*^2^ or Fisher’s exact tests for numerical, ordinal, and nominal data, respectively. The distribution of SSM values across the 253 patients included in the study was evaluated by the Kolmogorov–Smirnov test. Differences among the SSM measurements performed by different operators were analyzed via the Kruskal–Wallis with post hoc Dunn’s tests, followed by Holm *p*-value adjustments.

Since there were 20 independent measurements available for each patient, an assessment of the absolute error associated to different protocols with *n* records (*n* between 2 and 19) was performed by considering the median SSM across all the 20 measurements as reference. As an example, in order to assess a protocol designed with 5 measurements per patient, we calculated the median SSM value in each patient considering *n* = 5 records and then the absolute deviation (AD) from the median obtained from the 20 measurements. In order to have a robust estimation of the error, since in each examination the measurements are independent, the sampling of the *n* records from the 20 measurements of each patient was repeated 1000 times, considering then the median SSM values across the 253 patients, *i.e.*, med_ni_, with *n* = 2, …, 19 and *i* = 1, …, 1,000. At each iteration, the AD from the median SSM values across all the 20 measurements was calculated, *i.e.*, AD_ni_ =|med_ni_ − med_20i_| represents the distribution of the ADs from the reference median SSM values obtained by using a protocol with n measurements at the *i*th iteration. Since this distribution was derived from a population of 253 patients, here, we focused on the 95th percentile of the observed deviations. In the end, for each protocol with *n* records, the distributions of the estimations at 95% confidence were evaluated. The median and interquartile range (IQR) of AD distributions were reported in tables. Finally, we repeated the analyses by stratifying the patients in order to evaluate whether the error estimations might change considering patients with different etiologies (*i.e.*, CSPH and CLD) or at different ranges of SSM values (*i.e.*, < 25 kPa, 25–75 kPa, and > 75 kPa).

All analyses were performed using R programming language v. 4.1.2; *p*-values < 0.050 were considered statistically significant.

## Results

### Characteristics of the retrieved data

The anthropometric and spleen stiffness measurements for the cohort are reported in Table 1. The median age was 58 [46.0–66.5] years, and about 60% were males. The median body mass index was 28.4 (24.6–32.7) kg/m^2^. Overall, the median SSM was 52.5 [30.1–77.5] kPa, and it was significantly different between CSPH and CLD patients: medians at 68.6 [48.5–91.0] kPa and 30 [19.9–43.6] kPa, respectively (*p* = 0.042). In addition, the duration of the examinations (overall median at 4.4 [3.3–5.7] min) and the shear wave speed (overall median at 4.2 [3.2–5.1] cm/s) differed significantly between the two etiologies (*p* < 0.001).

**Table 1:** Characteristics of the cohort

Group	Total (*n* = 253)	CSPH (*n* = 153)	CLD (*n* = 100)	*p*-value
Sex				
Female	38.3% (97)	38.6% (59)	38% (38)	0.850
Male	58.5% (148)	60.8% (93)	55% (55)	
Unknown	3.2% (8)	0.7% (2)	7% (11)	
Age				
Median (IQR)	58 (46–66.5)	57 (46.5–66.5)	58 (44–64)	0.780
Body mass index				
Median (IQR)	28.4 (24.6–32.7)	28.9 (24.9–33.4)	27.4 (24–32.2)	0.063
Exam duration (min)				
Median (IQR)	4.4 (3.3–5.7)	4.1 (3.1–5.4)	4.8 (3.5–6.1)	0.042
SSM (kPa)				
Median (IQR)	52.5 (30.1–77.3)	68.6 (48.5–91)	30 (19.9–43.6)	< 0.001
Share wave speed (cm/s)				
Median (IQR)	4.2 (3.2–5.1)	4.8 (4–5.5)	3.2 (2.6–3.9)	< 0.001

Data are reported as median and IQR for continuous variables and as number and percentages for categorical variables

*CLD* Chronic liver disease, *CSPH* Clinically significant portal hypertension, *IQR* Interquartile range, *SSM* Spleen stiffness measurement

**Fig. 2:**
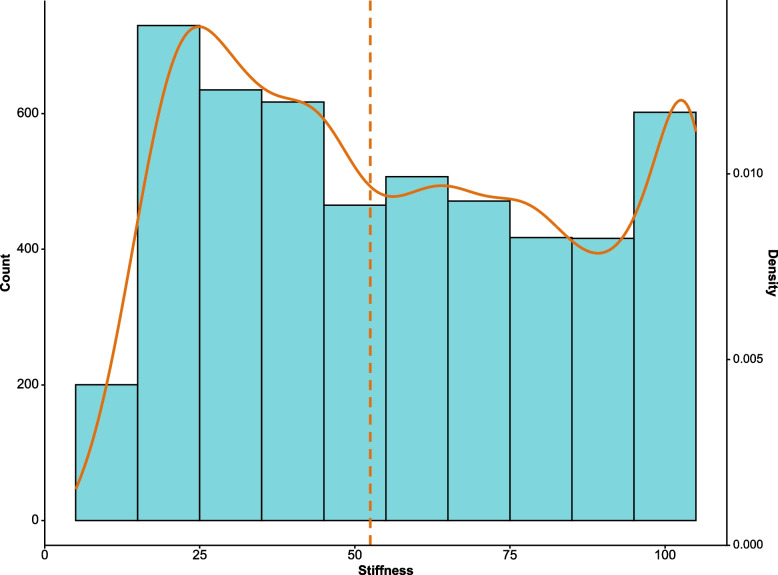
Histogram and density plot of the distribution of the median spleen stiffness values across the 20 scans. The dashed line indicates the median spleen stiffness value observed in the entire cohort

**Fig. 3:**
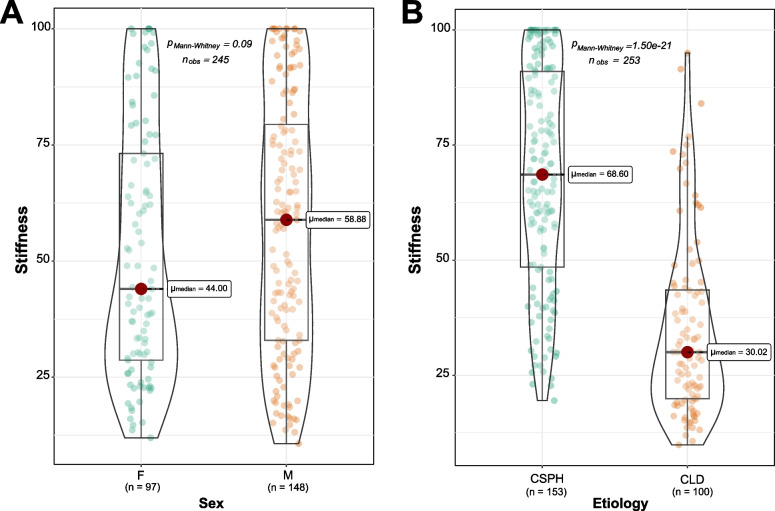
Comparison between the median spleen stiffness values stratifying the patients by sex and etiology. CLD, Chronic liver disease; CSPH, Clinically significant portal hypertension; *p*-values are from Wilcoxon rank sum test

The distribution of SSM values was non-normal (*D* = 0.07, *p* < 0.001, Fig. 2). No statistically significant differences were observed among the measurements performed by different operators (*p* = 0.320, Kruskall-Wallis test, Supplementary Fig. S1). The median SSM values across all the 20 scans per patient were not significantly different by sex (*p* = 0.090), but it is worth noticing that males tended to have higher SSM values (median at 58.9 [33.0–79.4] kPa *versus* 44 [28.7–73.2] kPa in females) and that the 7 cases at the maximum SSM scale excluded from the study were all males (Fig. 3a). On the other hand, significant differences were observed for the two etiologies: 68.6 [48.5–91] kPa *versus* 30 [19.9–43.6] kPa in CSPH and CLD patients, respectively (*p* < 0.001, Fig. 3b).

### Comparison between spleen stiffness measurements according to the number of scans

**Fig. 4:**
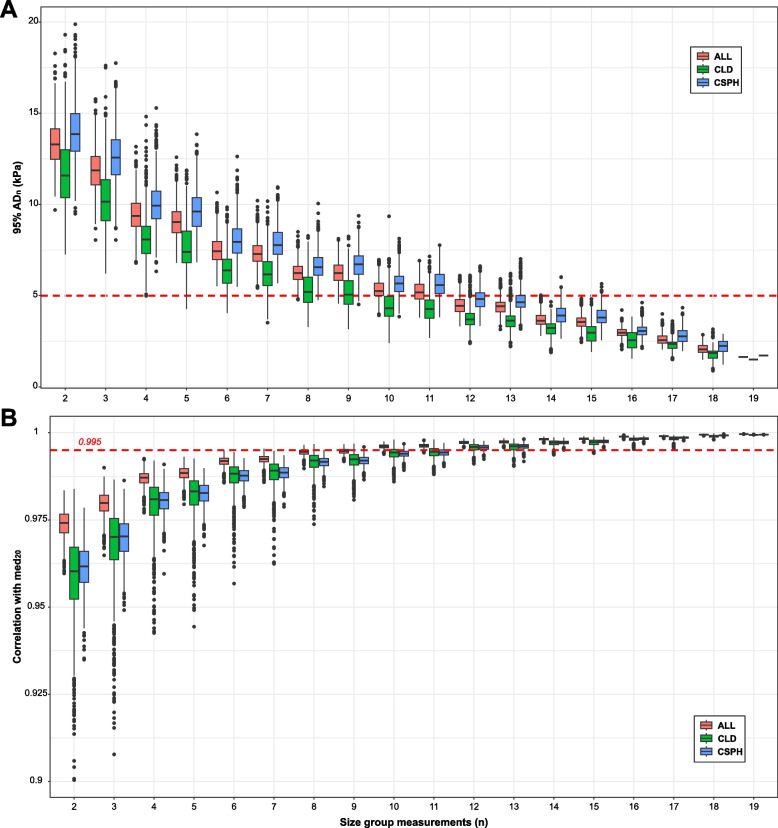
Distributions of the absolute deviations at 95% confidence at varying groups of measurements and stratifying by etiology. Distribution of 95% AD_*n*_ calculated at different protocols varying the number of measurements (*n*) from 2 to 19. AD, Absolute deviation; ALL, All patients; CLD, Chronic liver disease; CSPH, Clinically significant portal hypertension

**Fig. 5:**
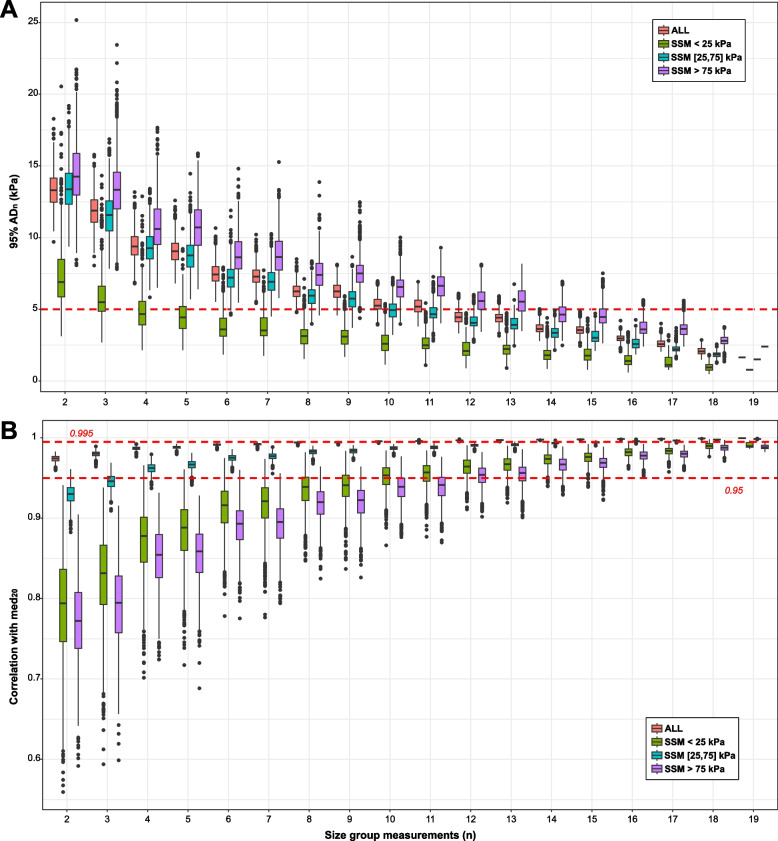
Distributions of the absolute deviations at 95% confidence at varying groups of measurements and stratifying by spleen stiffness ranges. Distribution of 95% AD_*n*_ calculated at different protocols varying the number of measurements (*n*) from 2 to 19. SSM ranges were subdivided into three groups: < 25 kPa, 25–75 kPa, and > 75 kPa. AD, Absolute deviation; ALL, All patients; SSM, Spleen stiffness measurement

Figure 4 shows the distribution of the AD_*n*_ (*n* = 2,…,19) considering both all the patients and dividing the cohort by etiology (*i.e.*, CSPH and CLD). Overall, 12 measurements seemed to be enough in order to have an AD within 5 kPa (*i.e.*, AD_12_ at 95% confidence with IQR between 4.1 and 4.8, Supplementary Table S1). It was possible to observe that the measurements from CSPH patients were burdened by higher variation than CLD patients (medians and IQRs reported in Supplementary Table S1). Considering both etiologies, in order to keep the ADs within 5 kPa, at least 14 measurements are recommendable (*i.e.*, AD_14_ at 95% confidence in CSPH patients with IQR between 3.6 and 4.2, Supplementary Table S1). Since the higher variability observed in CSPH patients suggests that the AD might depend on the SSM scale, we also displayed the distribution of the AD_*n*_ considering different ranges of SSM values: < 25 kPa, 25–75 kPa, and > 75 kPa (Fig. 5). For SSM values < 25 kPa, only 6 measurements were sufficient to obtain most of the ADs below 5 kPa (*i.e.*, AD_6_ at 95% confidence with IQR between 3.1 and 4.4, Supplementary Table S2). However, considering SSM values > 75 kPa, a higher number of measurements are required, at least 15 measurements in order to keep most of the ADs below or equal to 5 kPa (*i.e.*, AD_15_ at 95% confidence with IQR between 4 and 5, Fig. 5 and Supplementary Table S2).

## Discussion

Transient elastography is one of the best and most widely available tools to assess liver disease severity, allowing the risk stratification of patients with advanced chronic liver disease. Because of the close anatomic and physiologic connection of the spleen to the liver, there is a great interest in using the probe to also assess the spleen stiffness. Due to this interest and the inherent technical limitations of the 50-Hz probe, a new 100-Hz probe has been introduced into this field. In this retrospective evaluation of 365 SSM scans obtained using the novel FibroScan F630 Expert device with a spleen-specific 100-Hz probe, we found that, in general, the measurement of spleen stiffness stabilized after 12 measurements. However, for cases falling into higher SSM values (> 75 kPa), mostly compatible with CSPH, at least 15 measurements are required to decrease variability. These findings are similar to the reported liver stiffness measurements, whereby more than 10 scans during the procedure can overcome some of the technical limitations, including incorrect probe positioning [15].

Increased spleen stiffness has been linked to the onset of portal hypertension, due to the hypersplenism and the early modifications occurring in the splenic parenchyma in this clinical condition. In particular, high values of SSM (*i.e.*, > 75 kPa) have been linked to the presence of esophageal varices, which pose the risk for variceal bleeding. Accordingly, some studies suggest including SSM in the current algorithms to predict non-invasively the presence of esophageal varices, without unnecessary gastroscopies [9]. So far, the evaluation of spleen stiffness has been carried out using the commercially available FibroScan 50-Hz probe, which was specifically designed to measure the transient elastography of the liver. Unfortunately, these early studies have shown that the 50-Hz probe does not have the technical ability to accurately measure spleen stiffness [16] because the spleen parenchyma is stiffer and more superficially located than the liver. Therefore, the 100-Hz technology has been developed to overcome these limitations. As this device is only recently available, there is limited experience with little data available on the best procedures for the clinical evaluation of SSM measurements’ reliability. The manufacturer recommends performing ten measurements located at the spleen pole. However, in our cohort, the first ten measurements are burdened by a higher intraexamination variability (AD_15_ at 95% of confidence > 5 kPa for all patients, Figs. 4 and 5, Supplementary Tables S1 and S2). Our data indicated that the median SSM stabilizes after ten measurements, and at high SSM values (*i.e.*, > 75 kPa), the number of required measurements increases at 15 (Fig. 5 and Supplementary Table S2). The evaluation performed on our dataset shows that the measurement of the spleen is complicated and that more measurements are important to verify that the most accurate estimate is obtained in order to have a reproducible result. We observed that the measurements from CSPH patients were burdened by higher variation than CLD patients, and this might be explained by the higher SSM values observed in these patients: 68.6 [48.5–91] kPa *versus* 30 [19.9–43.6] kPa, respectively (Fig. 3b). In addition, no significant difference was observed between measurements performed by the three operators (*p* = 0.320, Kruskall-Wallis test, Supplementary Fig. S1), suggesting a good interoperator reproducibility of SSM. There are several reasons linked with the high intraexamination variability of the spleen scans observed by our study. The reduced dimensions of the spleen may prevent the correct positioning of the probe at the same point, leading to stiffness values that are captured in diverse portions of the spleen parenchyma. This sampling variability of transient elastography according to the probe positioning has been reported in liver stiffness examinations, with about 30% of variability according to the probe location [17]. Similarly, other studies reported an interoperator liver stiffness variability of 35% for at least one stage of liver fibrosis [18] and discrepancies of more than 10 kPa [19]. The data on the variations in spleen stiffness has not been as fully understood as the liver yet, and it is sometimes contradictory. For example, data extrapolated by SSM performed with shear wave elastography via acoustic radiation force impulse imaging, ARFI, showed that interobserver agreement was low (intraclass correlation coefficient 0.73), in particular for individuals with small spleen [20]. On the contrary, another study reported good interobserver agreement for 50-Hz SSM performed by transient elastography for either patients with chronic liver disease or healthy controls (intraclass correlation coefficient > 0.85) [21].

Despite the 253 patients included in the study, we did not find a significant difference in terms of SSM values between males and females (*p* = 0.090, Wilcoxon rank-sum test). Considering also the seven cases excluded because of the “saturated” SSM signal, the *p*-value would decrease at 0.03, suggesting a potential difference in spleen morphology between males and females. In the field of liver stiffness, the possibility to switch from M to XL probe has provided great advances, counteracting the technical limitations that may arise with the M probe for specific categories (*e.g.*, morbidly obese, deep location of the liver, increased subcutaneous thickness) [22, 23]. As already mentioned, the anatomically deeper location of the spleen, as compared to the liver, may be a major limitation for the suitability of SSM. The introduction of a spleen-specific XL probe could provide measurements that are more reliable by selecting the best exam according to the patient’s phenotype. Notably, a clinical trial is currently being conducted with the aim of an XL probe validation in the field of spleen stiffness [24].

Despite this study, a large number of scans were analyzed, and some limitations need to be highlighted. First, the lack of further clinical information in addition to the technical aspects prevents a translation of the results into the clinical setting, with particular regard to spleen size, which is acknowledged as one main determinant of spleen stiffness. Second, the data that were extracted refer only to successful examinations; hence, no data on the success rate of SSM in this cohort could be obtained. Importantly, the manufacturer does not suggest any IQR/median ratio values to assess the reliability of the exam conducted at 100 Hz. Despite our exams being all below the IQR/median ratio cutoff of 30% suggested for liver stiffness, we cannot draw any conclusions with regard to the precision of the measures. Finally, because this is a retrospective analysis, no data on intra- or interobserver agreement could be extracted.

In conclusion, because of the great interest in using spleen stiffness to assess portal hypertension within patients with advanced chronic liver disease, transient elastography could become as highly used as liver stiffness measurements in this patient group. Therefore, we believe that it is important to understand the technical abilities and limitations of this novel device and what steps can be taken to reduce variability and improve the measurement of spleen stiffness. Based on our results, we suggest that the number of 100-Hz SSM by transient elastography should exceed 12 measurements per examination. The current study collected up to 20 measurements per patient, and the average examination time in this cohort was 4:30 min, which does not significantly affect the feasibility of the spleen evaluation. The slightly but significantly longer duration of the exam in CLD patients may be due to the spleen size, which is within the normal range and thus less readily reachable, as compared to the larger size seen in CSPH. However, performing at least 15 measures leads to the greatest stabilization in the variability in order to limit the potential technical difficulties that may arise due to the intrinsic features of the spleen. We believe that the advent of the 100-Hz probe is a step forward for this technique and that the proper usage of the device will produce reliable and valuable data for patient care.

### Supplementary Information

**Additional file 1: Supplementary Table 1.** Distributions of the absolute deviations at 95% of confidence at varying groups of measurements and stratifying by etiology. Median and inter-quartile range (IQR) of absolute deviation at 95% confidence are reported for different protocols varying the number of measurements n (*n* = 2,…,19). CSPH: clinically significant portal hypertension; CLD: chronic liver disease. **Supplementary Table 2.** Distributions of the absolute deviations at 95% of confidence at varying groups of measurements and stratifying by SSM ranges. Median and inter-quartile range (IQR) of absolute deviation at 95% confidence are reported for different protocols varying the number of measurements n (*n* = 2,…,19). SSM ranges were divided into 3 groups: < 25 kPa, [25–75] kPa and > 75 kPa. **Supplementary Fig. S1.** Comparison among median SSM values stratifying the examinations by operator. *p*-value for comparison of multiple groups from Kruskal-Wallis test; *p*-values for pairwise comparisons from Dunn’s test, with Holm’s adjustment.

## Data Availability

Aggregated, anonymized data is available from the corresponding author upon reasonable request and when in accordance with GDPR.

## Abbreviations

AD: Absolute deviation
CLD: Chronic liver disease
CSPH: Clinically significant portal hypertension
IQR: Interquartile range
SSM: Spleen stiffness measurement
