# Supplementary material for: Variability of transient elastography-based spleen stiffness performed at 100 Hz
Source: Eur Radiol Exp. 2023 Dec 12;7:79. doi: 10.1186/s41747-023-00393-2 (PMC10716091; doi:10.1186/s41747-023-00393-2)
Supplement: Supplementary file 1 — Additional file 1: Supplementary Table 1. Distributions of the absolute deviations at 95% of confidence at varying groups of measurements and stratifying by etiology. Median and inter-quartile range (IQR) of absolute deviation at 95% confidence are reported for different protocols varying the number of measurements n (n = 2,…,19). CSPH: clinically significant portal hypertension; CLD: chronic liver disease. Supplementary Table 2. Distributions of the absolute deviations at 95% of confidence at varying groups of measurements and stratifying by SSM ranges. Median and inter-quartile range (IQR) of absolute deviation at 95% confidence are reported for different protocols varying the number of measurements n (n = 2,…,19). SSM ranges were divided into 3 groups: < 25 kPa, [25–75] kPa and > 75 kPa. Supplementary Fig. S1. Comparison among median SSM values stratifying the examinations by operator. p-value for comparison of multiple groups from Kruskal-Wallis test; p-values for pairwise comparisons from Dunn’s test, with Holm’s adjustment. [file 41747_2023_393_MOESM1_ESM.docx]

**Variability of transient elastography-based spleen stiffness performed at 100 Hz.**

**ELECTRONIC SUPPLEMENTARY MATERIAL**

| **Number of measurements** | **ALL** | **CLD** | **CSPH** |
| --- | --- | --- | --- |
| 2 |  |  |  |
| Median (IQR) | 13.3 (12.5-14.1) | 11.6 (10.4-13) | 13.9 (12.9-15) |
| 3 |  |  |  |
| Median (IQR) | 11.9 (11.1-12.6) | 10.2 (9.1-11.4) | 12.6 (11.6-13.6) |
| 4 |  |  |  |
| Median (IQR) | 9.4 (8.8-10.1) | 8.1 (7.3-8.8) | 9.9 (9.2-10.7) |
| 5 |  |  |  |
| Median (IQR) | 9 (8.5 -9.6) | 7.4 (6.8-8.5) | 9.6 (8.8-10.4) |
| 6 |  |  |  |
| Median (IQR) | 7.4 (7-8) | 6.4 (5.7-7) | 8 (7.3-8.7) |
| 7 |  |  |  |
| Median (IQR) | 7.3 (7-7.7) | 6.2 (5.6 -6.9) | 7.8 (7.3-8.5) |
| 8 |  |  |  |
| Median (IQR) | 6.2 (6-6.6) | 5.2 (4.6-6) | 6.6 (6.1-7.1) |
| 9 |  |  |  |
| Median (IQR) | 6.2 (5.8-6.7) | 5.1 (4.5-5.8) | 6.7 (6.2-7.2) |
| 10 |  |  |  |
| Median (IQR) | 5.3 (5-5.7) | 4.3 (3.9-5) | 5.7 (5.2-6.1) |
| 11 |  |  |  |
| Median (IQR) | 5.2 (4.8-5.6) | 4.3 (3.8-4.8) | 5.6 (5.1-6.2) |
| 12 |  |  |  |
| Median (IQR) | 4.5 (4.1-4.8) | 3.7 (3.4-4) | 4.8 (4.4-5.2) |
| 13 |  |  |  |
| Median (IQR) | 4.4 (4.1-4.6) | 3.6 (3.3-3.9) | 4.6 (4.4-5) |
| 14 |  |  |  |
| Median (IQR) | 3.6 (3.4-3.9) | 3.2 (2.9-3.5) | 3.9 (3.6-4.3) |
| 15 |  |  |  |
| Median (IQR) | 3.6 (3.3-3.8) | 3 (2.5-3.3) | 3.8 (3.5-4.2) |
| 16 |  |  |  |
| Median (IQR) | 3 (2.8-3.1) | 2.6 (2.1-3) | 3.1 (2.9-3.3) |
| 17 |  |  |  |
| Median (IQR) | 2.6 (2.4-2.8) | 2.4 (2.1-2.5) | 2.8 (2.5-3.1) |
| 18 |  |  |  |
| Median (IQR) | 2.1 (1.9-2.3) | 1.9 (1.6-2) | 2.2 (2-2.5) |
| 19 |  |  |  |
| Median (IQR) | 1.6 (1.6-1.6) | 1.5 (1.5-1.5) | 1.7 (1.7-1.7) |

| **Number of measurements** | **ALL** | **SSM <25kPa** | **SSM ≥25kPa & ≤ 75kPa** | **SSM <75kPa** |
| --- | --- | --- | --- | --- |
| 2 |  |  |  |  |
| Median (IQR) | 13.3 (12.5-14.1) | 6.9 (5.9-8.5) | 13.4 (12.3-14.5) | 14.3 (13-15.9) |
| 3 |  |  |  |  |
| Median (IQR) | 11.9 (11.1-12.6) | 5.5 (4.9-6.6) | 11.6 (10.5-12.6) | 13.3 (12-14.6) |
| 4 |  |  |  |  |
| Median (IQR) | 9.4 (8.8-10.1) | 4.7 (3.9-5.6) | 9.3 (8.5-10) | 10.6 (9.5-12) |
| 5 |  |  |  |  |
| Median (IQR) | 9 (8.5 -9.6) | 4.4 (3.7-5.2) | 8.8 (7.9 -9.5) | 10.7 (9.5-11.9) |
| 6 |  |  |  |  |
| Median (IQR) | 7.4 (7-8) | 3.6 (3.1-4.4) | 7.2 (6.6-7.8) | 8.6 (7.8-9.7) |
| 7 |  |  |  |  |
| Median (IQR) | 7.3 (7-7.7) | 3.5 (3.2-4.4) | 6.9 (6.3-7.6) | 8.6 (7.7-9.8) |
| 8 |  |  |  |  |
| Median (IQR) | 6.2 (6-6.6) | 3.1 (2.6-3.6) | 6 (5.4-6.4) | 7.4 (6.7-8.2) |
| 9 |  |  |  |  |
| Median (IQR) | 6.2 (5.8-6.7) | 3.1 (2.6-3.5) | 5.7 (5.2-6.2) | 7.5 (6.9-8.1) |
| 10 |  |  |  |  |
| Median (IQR) | 5.3 (5-5.7) | 2.6 (2.1-3.2) | 5 (4.5-5.4) | 6.6 (5.9-7) |
| 11 |  |  |  |  |
| Median (IQR) | 5.2 (4.8-5.6) | 2.5 (2.3-3) | 4.7 (4.4-5.1) | 6.6 (5.9-7.3) |
| 12 |  |  |  |  |
| Median (IQR) | 4.5 (4.1-4.8) | 2.1 (1.8-2.7) | 4.1 (3.8-4.5) | 5.6 (5-6.2) |
| 13 |  |  |  |  |
| Median (IQR) | 4.4 (4.1-4.6) | 2.2 (1.9-2.5) | 3.9 (3.6-4.4) | 5.5 (4.9-6.3) |
| 14 |  |  |  |  |
| Median (IQR) | 3.6 (3.4-3.9) | 1.8 (1.5-2.1) | 3.4 (3.1-3.7) | 4.6 (4.1-5.2) |
| 15 |  |  |  |  |
| Median (IQR) | 3.6 (3.3-3.8) | 1.8 (1.5-2.2) | 3 (2.8-3.5) | 4.5 (4-5) |
| 16 |  |  |  |  |
| Median (IQR) | 3 (2.8-3.1) | 1.4 (1.1-1.8) | 2.6 (2.3-2.9) | 3.6 (3.3-4.1) |
| 17 |  |  |  |  |
| Median (IQR) | 2.6 (2.4-2.8) | 1.1 (1-1.6) | 2.2 (2.1-2.4) | 3.6 (3.2-3.9) |
| 18 |  |  |  |  |
| Median (IQR) | 2.1 (1.9-2.3) | 0.9 (0.75-1.1) | 1.9 (1.7-2) | 2.8 (2.6-3) |
| 19 |  |  |  |  |
| Median (IQR) | 1.6 (1.6-1.6) | 0.8 (0.8-0.8) | 1.5 (1.5-1.5) | 2.4 (2.4-2.4) |

**Supplementary Table 2. Distributions of the absolute deviations at 95% of confidence at varying groups of measurements and stratifying by SSM ranges.** Median and inter-quartile range (IQR) of absolute deviation at 95% confidence are reported for different protocols varying the number of measurements n (n=2,…,19). SSM ranges were divided into 3 groups: <25 kPa, [25-75] kPa and >75 kPa.


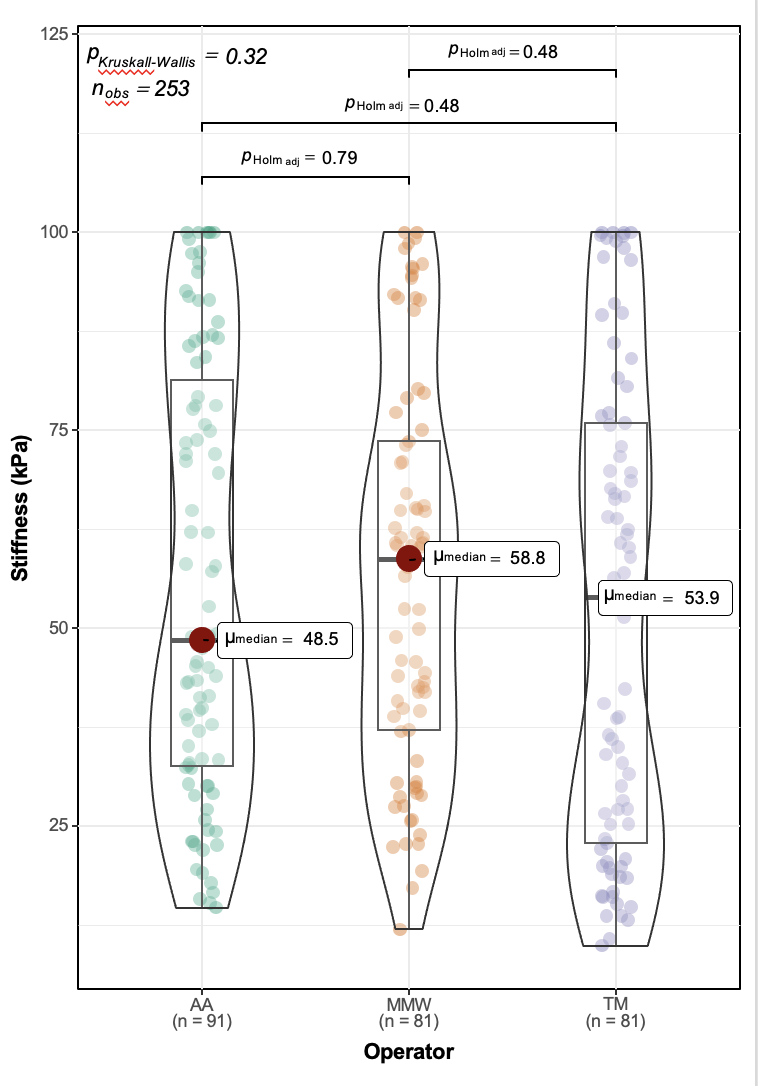


**Supplementary Fig. S1**. Comparison among median SSM values stratifying the examinations by operator. P-value for comparison of multiple groups from Kruskal-Wallis test; p-values for pairwise comparisons from Dunn’s test, with Holm’s adjustment
